# Bayesian Network Modelling of ATC Complexity Metrics for Future SESAR Demand and Capacity Balance Solutions

**DOI:** 10.3390/e21040379

**Published:** 2019-04-08

**Authors:** Victor Fernando Gomez Comendador, Rosa Maria Arnaldo Valdés, Manuel Villegas Diaz, Eva Puntero Parla, Danlin Zheng

**Affiliations:** 1 Air Transport and Airports Department, School of Aerospace Engineering, Technical University of Madrid (UPM), 28040 Madrid, Spain; 2 ATM Research and Development Reference Centre (CRIDA), 28022 Madrid, Spain

**Keywords:** Bayesian networks, complexity, uncertainty, TBO, SESAR Capacity Management, DCB, DAC, FCA

## Abstract

Demand & Capacity Management solutions are key SESAR (Single European Sky ATM Research) research projects to adapt future airspace to the expected high air traffic growth in a Trajectory Based Operations (TBO) environment. These solutions rely on processes, methods and metrics regarding the complexity assessment of traffic flows. However, current complexity methodologies and metrics do not properly take into account the impact of trajectories’ uncertainty to the quality of complexity predictions of air traffic demand. This paper proposes the development of several Bayesian network (BN) models to identify the impacts of TBO uncertainties to the quality of the predictions of complexity of air traffic demand for two particular Demand Capacity Balance (DCB) solutions developed by SESAR 2020, i.e., Dynamic Airspace Configuration (DAC) and Flight Centric Air Traffic Control (FCA). In total, seven BN models are elicited covering each concept at different time horizons. The models allow evaluating the influence of the “complexity generators” in the “complexity metrics”. Moreover, when the required level for the uncertainty of complexity is set, the networks allow identifying by how much uncertainty of the input variables should improve.

## 1. Introduction

The foreseen high level of air traffic demand expected in Europe in the coming years can only be managed by a new and advance Air Traffic Management (ATM) System. This new and complex ATM system developed by the European Union and European industry is known as SESAR 2020 (Single European Sky ATM Research), which is the technological pillar of the Single European Sky, and a key enabler of the EU Aviation Strategy.

The most relevant SESAR 2020 solutions [1,2] dealing with future Capacity Management processes are Dynamic Airspace Configuration (DAC) [3,4] and Flight Centric Air Traffic Control (FCA) [5]. Both of them are applied within the Trajectory Based Operations (TBO) environment. TBO [6] is the exchange, maintenance and use of consistent aircraft trajectory and flight information for collaborative decision-making on the flight. TBO relies on the four-dimensional (4D) trajectory concept [7]. SESAR defines the 4D trajectory as a set of consecutive segments linking published waypoints and/or pseudo waypoints computed by air or ground tools (FOC—Flight Operation Centre-system, aircraft FMS—Flight Management System, ground Trajectory Predictor) to build the lateral transitions and the vertical profiles. Because of the TBO related processes, the information available in the different phases of the planning process improves consistently the trajectories data managed by different stakeholders and decreases flight data inconsistencies.

The DAC process aims to identify an optimised airspace configuration based on the complexity predicted, ATCos (Air Traffic Controllers) availability and defined performance targets. In this regard, the number of controlled sectors and their shape will adapt to the traffic situation. It comprises two types of processes:

(1) Sector Design processes supported by automation to delineate airspace structures and elementary sectors according to DAC local implementation.
(2) Sector management processes produce the sector configuration to match the traffic for a given period. Sector Management considers multiple criteria and constraints in the search for an optimal solution, with complexity assessment being the most relevant [8].

In terms of FCA concept, controllers will not be longer in charge of managing the entire traffic within a given sector. Instead, controllers will be responsible for a certain number of aircraft throughout their flight segment within the airspace under their responsibility. FCA will dissolve sector boundaries in order to obtain large size airspace such as Functional Airspace Blocks (FAB), and jurisdiction will be shared among several controllers within the same airspace. Two traffic allocation schemes have been defined: dynamic allocation and static allocation. Both are based on the traffic information available and the complexity level, which will determine the effects on controllers’ workload.

Both solutions, DAC and FCA, rely on complexity assessment of traffic flows and on integrating predicted workload function and confidence index. For this reason, complexity assessments process [8], methods [9,10,11,12,13,14,15] and metrics [16,17,18] become one of the main constraints to deal with the growing demand and increasing airspace capacity. SESAR defined complexity as the “…measure of the difficulty that a particular traffic situation will present to an air traffic controller…” [9]. This view of complexity is linked to the behavioural complexity (the observable activity of the controller to manage a traffic situation) and the cognitive complexity (the mental effort that a controller will require to comprehend and implement the control activity).

However, current methods and metrics for complexity evaluation are not adapted to the particular needs of DAC and FCA, as they do not properly take into account trajectory uncertainty. Uncertainty over the agreed TBO trajectories, and complexity uncertainty as an unavoidably consequence, will affect the effectiveness of the Capacity Management if it is not properly managed. Moreover, despite the fact that TBO allows obtaining reliable information related to trajectory uncertainty, thanks to the better reliability of the available trajectory information, the effect of TBO and trajectory uncertainties on complexity calculation has not yet been properly studied [10]. The assessment and definition of a proper use and integration of this trajectory uncertainty information within complexity calculation will significantly enforce effectiveness of the SESAR Capacity Management.

In this context, this paper addresses the challenge of exploring how the uncertainties associated with the agreed TBO trajectory will influence the quality of the predictions of complexity of traffic demand and the effectiveness of DCB processes, DAC and FCA, regarding airspace management.

To date, uncertainty within TBO has been usually quantified by means of uncertainty propagation techniques, where a statistical realization of a system’s input parameters is propagated through a numerical model to construct the statistics of the system’s outputs. The COPTRA (Combining Probable TRAjectories) project [11] considered the inputs to the trajectory prediction process as probabilistic distributions that define the nominal value of a variable and the associated uncertainty. The Polynomial Chaos Expansion [12] method determines the evolution of probabilistic uncertainty in a dynamical system by means of polynomials functions. The Mixed-integer Linear Programming [13] methods minimise a linear function combination of uncertainty sources vectors, subject to constraints. The Linear Regression Approach [14] has been used to estimate the uncertainty in trajectory crossing time at a sector boundary (entry or exit) and the uncertainty in sector traffic load [15]. Monte Carlo Simulations [16] have been used to estimate the probability of compliance with tolerance windows. Ellipsoid trajectory uncertainty modelling [17] relies on the direct characterisation of trajectory uncertainty, from real statistical aircraft data, without analysing the sources of such uncertainty. Queuing models [18] characterise uncertainty in aircraft operations by probability distributions of service times in a queuing system.

All the previous approaches consider unidirectional propagation of uncertainties. These methods work well for sensitivity studies, but present limitations when data are available at the output level or at some intermediate stage within the analysis. Limitations are even greater when data are not available for the characterisation of the uncertainty of a variable, and all the information can only be elicited from expert knowledge. In such cases, the consequent backward propagation of information needs to be properly modelled. Limitations are not surmountable when there is no detailed knowledge of the phenomena to construct a mathematical model of the process outcomes. Therefore, this work proposes an alternative robust technique, which is Bayesian Network (BN) modelling of the uncertainty propagation in the calculation of ATM complexity.

The Bayesian models relate complexity generators and their influence in ATM complexity for the two operational concepts, DAC and FCA, at various timeframe horizons. The long-term horizon covered from 5 years up to 6 months before the day of the operations. The general high-level goal of network operations planning in this period was to translate the ATM Network Strategy Plan into a European Network Operations Plan. During this time horizon, different data (Routing Preference (historical data), Priorities, existing airspace structure, airspace availability, etc.) were collected to improve the accuracy of traffic demand forecasts. Medium term time horizon started months before to the day of operations and extended to one week before the day of the operation. The Short term time horizon concerned the day of operation. In the Short-term time horizon, the available information allowed to prepare daily plans in collaboration with all stakeholders.

The aim of the networks was to help to identify the relevant variables in the process, and to understand the causal relationships and interdependences between factors influencing the complexity and the uncertainties associated to those factors [19,20,21]. The outcome of these models was two-fold.

- On one side, they will help to identify what complexity generators need to be properly taken into account and analysed by the complexity algorithms and metrics adapted to the DAC and FCA concepts. Among the set of variables considered complexity generators, the BN will help to highlight which ones are expected to have a High (H), Medium (M), or Low (L) influence in the complexity outcome itself. The future complexity algorithms and metrics might take this outcome as an indication of which variables, because of their influence on complexity, need to be carefully addressed by the metrics and algorithms.
- At the same time, the networks will help to identify whether the a-priori uncertainty probability distribution of each one of those variables will be compatible with maintaining the uncertainty of complexity outcome at a “Low” or “Medium” state; or, conversely, which ones will need to reduce its level of uncertainty. The future complexity algorithms and metrics might take this outcome as an indication of the uncertainty they might expect for each complexity generator (High, Medium or Low), and according to that decide the best approach to characterise and measure this variable in each scenario.

Bayesian networks have become a popular and well-spread modelling and analysis technique in aviation during the last decades. They are particularly useful to capture and analyse causality and influence’s relationships, and they are a convenient and coherent way to represent uncertainty in uncertainty models. In particular, they have the capacity to model propagation of multi-directional uncertainty forward and backward; thus, they are a useful tool for both predicting the performance of a system or diagnosing the causes of a certain system outcome [20,21].

### Assessment of Uncertainty Through Bayesian Networks

Most decisions in system design and assessment imply judgement under uncertainty. Sometimes these decisions are taken under the assumption that the values of the parameters describing the system performance are equal to their estimates. However, this postulation is only valid as long as there is sufficient data or precise expertise for an accurate estimation of the system parameters [22]. This is not the case in many situations, particularly when the system, product or process is new and only tiny measurable information about its performance is accessible. Uncertainty often arises from incomplete, inaccurate or imprecise models or from data collection during the safety compliance assessment.

There are several approaches to the concept of uncertainty [23,24,25]. Uncertainty is often understood as a “state of knowledge” [26]. Ayyub [27] describes it in terms of knowledge imperfection due to intrinsic shortages of knowledge acquisition. Walker [28] expresses uncertainty as “any departure from the unachievable ideal of complete determinism”. Aven [29] defines it as a “…lack of understanding about the behaviour and outcomes of a system, and discernible magnitudes”.

Although there is a wide variety of definitions for the concept of uncertainty, the common element in all of them is the idea of imperfect or incomplete knowledge of a system and its performances because of deficiencies in discernible information and observable data [30,31].

Uncertainty refers to the stochastic behaviour of a system and to the uncertain values of the parameters that describe it. Consequently, it might have either and aleatory or an epistemic origin. Aleatory uncertainty refers to the natural and inherent variation of the physical phenomena and their magnitudes, while epistemic uncertainty refers to the incomplete or limited knowledge of the values of parameters explaining the system behaviour [32,33].

Aven discusses the treatment of uncertainties in risk assessment for practical decision-making [24]. The relevance of this topic has also been highlighted by Leonong et al. in their paper on the safety assurance of socio-technical systems [32], by Zhihuang and Scott in their study on robust design [33] and by Percy in his work on reliability [34].

A systematic approach for dealing with uncertainties in decision-making is possible through Bayesian reasoning. Bayesian inference is a scientific formalism that helps decision-makers to select an appropriate path in relation to the acceptance of a system against its results, particularly when there is uncertainty about the consequences of the decision. Bayesian decision theory could also take into account the preferences of the decision-makers, experts’ knowledge regarding the uncertainties, the structure and complex relationships between the set of relevant factors, and the consequences of the decisions to be made.

Bayesian methods have been applied to decision-making problems under uncertainty in a variety of fields. They provide a formal probabilistic method for addressing the issue, a user friendly visual interface and efficient computational tools for analysing consequences and risks. In order to take advantage of data and information, Yu Feng et al [35], have implemented discrete time Bayesian networks and dynamic Bayesian networks to model dynamic fault trees and event tress in safety assessments. Moreover, some authors [36] have used Bayesian belief networks, combined with fuzzy logic, to represent dependences between events, update probabilities and represent uncertain knowledge in a variety of accident scenarios. Ancel et al. modelled the risk involved in flight crew integration with automation Bayesian models [37]. Prabhakaran illustrated the use of Bayesian Networks for System Safety Analysis of Critical System Applications [38]; Eleye-Datubo models risks in marine and offshore safety assessment in a Fuzzy-Bayesian Network [39]; Villa and Cozzani used Bayesian Networks to quantitatively assess the performance of safety barriers [40]. In [21], hierarchical Bayesian models based on binomial and Poisson distributions were used to model different uncertainties in air safety occurrences. In [41], the authors used BN quantify resilience of infrastructure systems in transportation networks as a function of absorptive, adaptive and restorative capacities.

Hosseini and Braker have developed a line of though by applying Bayesian Networks to the analysis of supply chain under uncertainty. In [42], they proved the benefits of BN to handle expert evidence and to perform sensitivity and propagation analyses and to understand the causal relationships among variables in the context of supplier evaluation and selection. In [43], the same authors dug into the design of resilient supply chains which are capable of withstanding and recovering rapidly from disruptive events.

Some attempts have been made to analyse the uncertainty problem in the assessment of safety performances. Mechri et al. [44] have proposed Bayesian networks that address the imperfect knowledge concern through a factor of Common Cause Failure (CCF) defined as triangular fuzzy numbers that express expert’s uncertainties about CCF values. This work illustrated how the CCF uncertainties are propagated through the Bayesian network inducing uncertainty to the values of the safety system performance. In [45], the authors presented a sociotechnical model based on Bayesian belief networks (BBN) for investigating multi-crew civil aircraft accident rate. The model captures the impact of airline strategies. This approach allowed quantitative safety assessment despite the low probability of the accidents rates. It explained the six or seven orders of magnitude gap between in-flight pilots’ error and the accident rate.

Bayesian networks have also been used for safety assessments in ATM because of its power to express knowledge and its capability to reason under uncertain environment. In [46], the authors apply Bayesian inference to the safety assessment of RPAS. The model was demonstrated to be able to deal with complicated logic relationships and several expert opinions.

Following the previous discussion, Bayesian networks have been selected in this case to assess the uncertainty propagation for complexity in ATM because their following capabilities:

- Bayesian networks are useful to capture and analyse causality and influence relationships. They are very efficient at propagating the uncertainty and updating the system with new data in the network. They are also applicable when system structures are too complex and offer an intuitive and efficient way of representing sizable domains, making modelling of complex systems practical.
- Bayesian networks are primarily used to update the probability distribution over the states of a hypothesis variable: a variable that is not directly observable. This probability distribution then helps a decision-maker in deciding upon an appropriate course of action [47].
- Bayesian networks provide a convenient and coherent way to represent uncertainty in uncertain models and are increasingly used for representing uncertain knowledge. They are used to model uncertainty and reason about it in both a qualitative and a quantitative manner.
- Due to the conditional dependence relationships of the variables within the network, BNs offer the ability to either predict or diagnose (i.e., they can determine effects and causes). Bayesian Networks are used to model multi-directional uncertainty forward and backward propagation.
- Bayesian networks allow for qualitative cause and effect assessment as well as for and quantitative updated on the probability distribution of non-observable variables. Qualitative analysis: given a scenario, a Bayesian network depicts graphically the cause and effect relationship between various elements of the scenario. Quantitative analysis: updates probability distributions. Given a situation and a prior probability distribution over a hypothesis variable that represents possible courses of action, Bayesian network provides the capability to update this probability distribution when fresh data and information are obtained.

## 2. Materials and Methods: Construction of a Bayesian Network for Assessing Causality and Modelling Uncertainty in ATM Through Bayesian Networks

**Figure A1:**
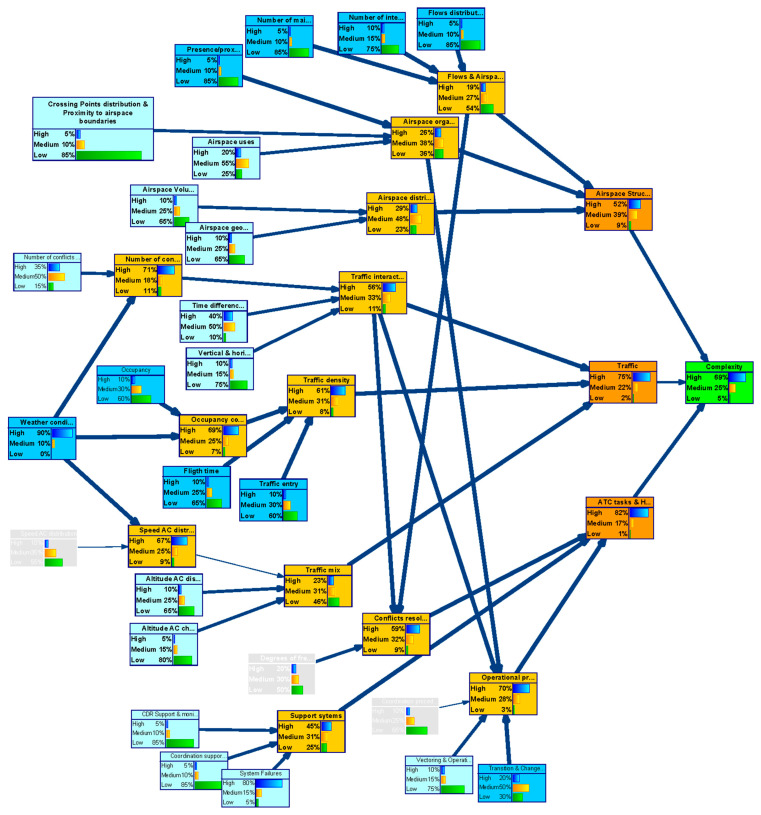
Forward inter-causal analysis (DAC medium term model).

**Figure A2:**
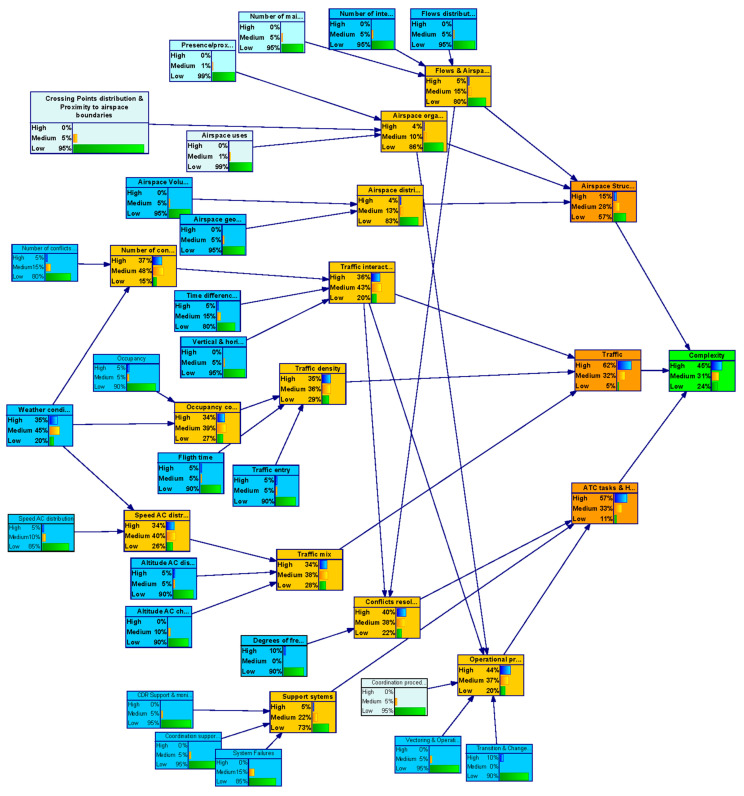
Forward inter-causal analysis (DAC short term model).

**Figure A3:**
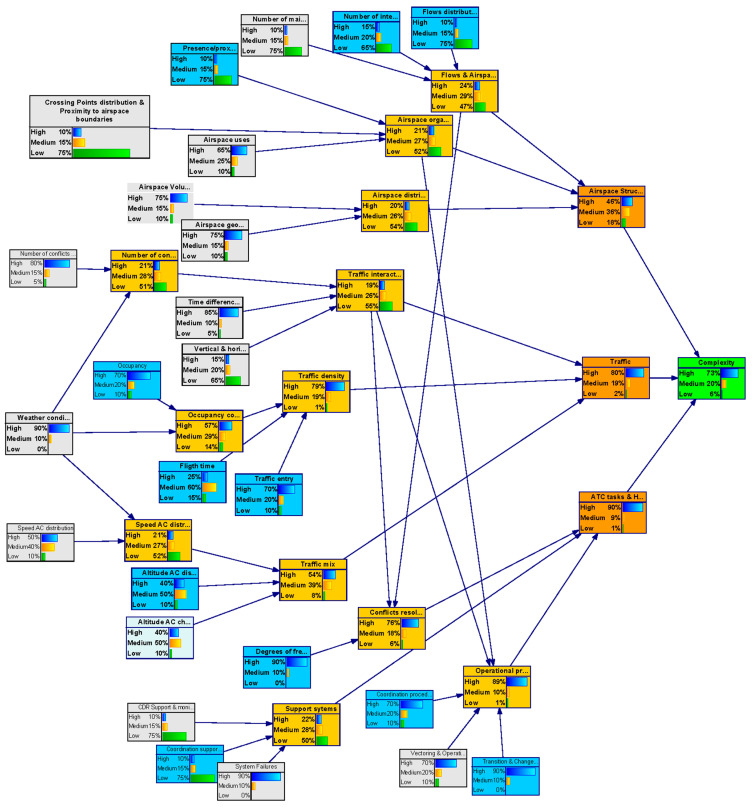
Forward inter-causal analysis (FCA long term model).

**Figure A4:**
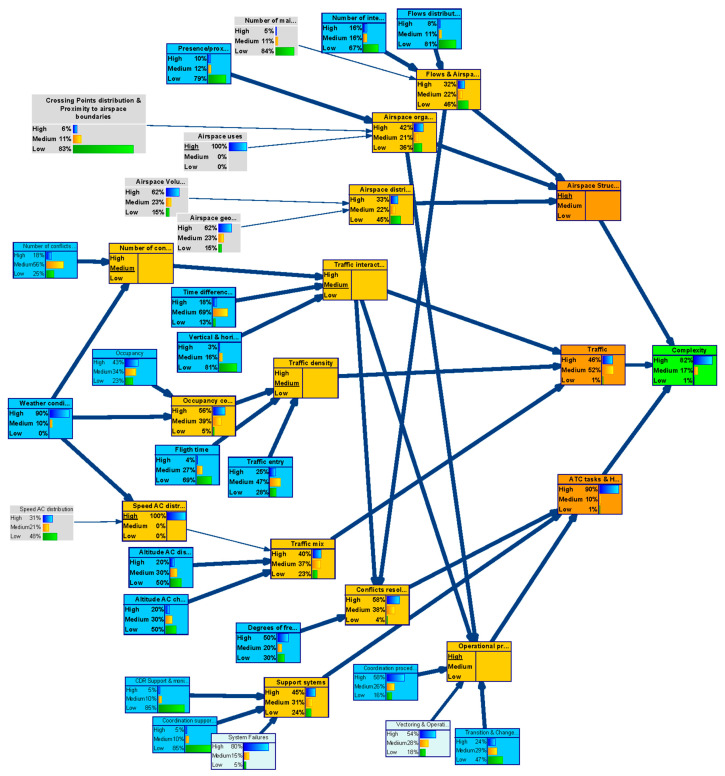
Forward inter-causal analysis (FCA medium term model).

**Figure A5:**
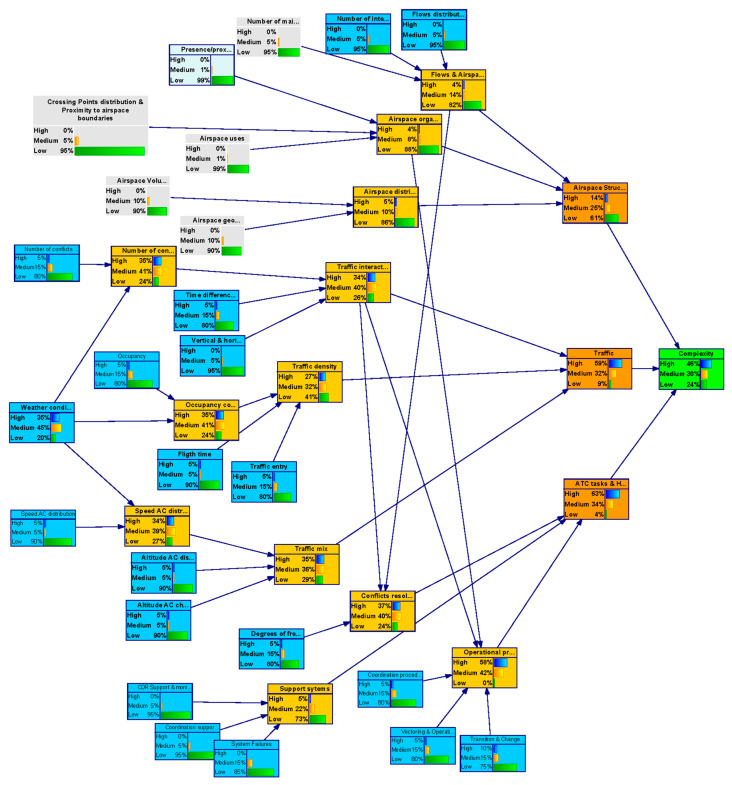
Forward inter-causal analysis (FCA short term model).

A BN can be constructed either manually, based on knowledge and experiences acquired from previous studies and literature, or automatically from data. Our models aimed to capture the main complexity generators as well as the relationships between them, and to explain how the level of uncertainty about the values of each complexity generator propagates and generates uncertainty in the final value of complexity. Initially, a generic model was built based on the variables and causal relationship identified from the literature survey and by the experts participating in the project. The generic Bayesian model was further adapted to each one of the operational concepts consider in the project, DAC and FCA, and to each one of its time horizons, long-term, medium term and short term/execution. In total, seven Bayesian models were elicited, as indicated in Table 1. The first model (complexity for DAC in the long term horizon) is illustrated and discussed through the text. The remaining 5 models are presented in Appendix A on Figure A1, Figure A2, Figure A3, Figure A4 and Figure A5.

The BN models have been built and validated following a process model called Knowledge Engineering of Expert–based Bayesian Network (EKEBN) [48]. This process comprises three main steps: (1) structure building; (2) uncertainty quantification; and (3) model validation. The process is iterative until a complete BN is built and validated.

Structure building consisted in the identification of the variables and causal relationships and the states or values that each variable could take. The BN’s structure was refined through an iterative process. The structure has been validated by two combined approaches. Experts have been divided in two groups who assessed iteratively the models to detect inconsistencies and discrepancies. Additionally, representativeness of the variables, causal relationships and possible states have been independently contrasted with the available literature. When no evidences sustained the expert’s elicitation, experts have been asked to refine their appreciation. Additionally, consistency checks have been applied to verify that all variables are clearly defined and have unique meanings, that all relevant variables have been included and that all the states are exhaustive and exclusive.

Uncertainty quantification deals with the conditional probabilities that quantify the relationships between variables. Conditional probability tables (CPTs) were obtained via expert’s elicitation as well from data and results in existing literature. Finally, the model validation checks the BN resulting from the two previous steps and determines whether it is necessary to revisit any of the previous steps. Validation followed a Model Walkthrough approach. It consisted of the use of real case scenarios that were prepared by domain experts to assess the predictions provided by the BN model. It determined up to what extent BN predictions correspond to the predictions experts would have made. Case scenarios have been elaborated from available studies applying current Air Traffic complexity models. A model walkthrough provides a subjective assessment of how good a model is perceived at providing or measuring what is expected of it [49]. If the results provided by the Bayesian network seem plausible for different hypothetical scenarios, this is interpreted as the goodness of the BN model’s output to capture the expectative of the stakeholders. We have also verified the adequacy of the outcome with case scenarios from literature not used for model building. In case of discrepancies, the model was recalibrated.

**Figure 1:**
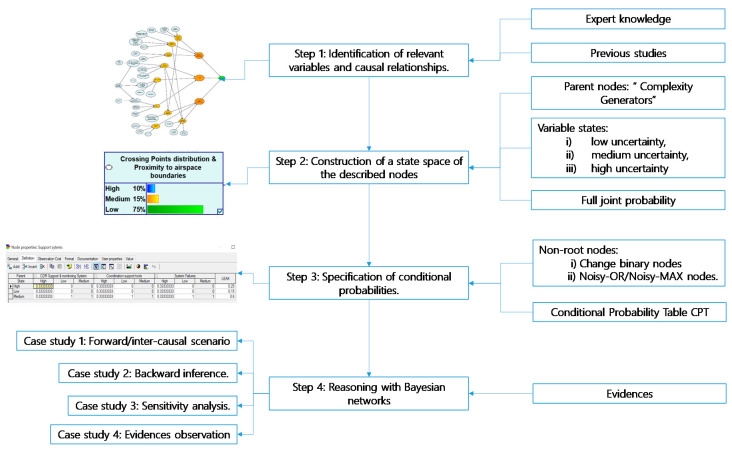
Steps in the construction of a generic Bayesian Network for complexity. (CPT: Conditional Probability Tables).

Figure 1 summarizes the main steps followed for building a Bayesian Network. Each step is explained in the following sections of this chapter, using as illustrative example the construction of the generic complexity Bayesian network.

The network structure and experts’ estimates for probability tables (PTs) and conditional probability tables (CPTs) were elicited on paper. A variety of elicitation formats for the parameterization of BNs is available on the literature. For this project a group of 20 Air Traffic Management experts were carefully selected. Experts’ estimates were treated equally and no weighted averages were calculated. When discrepancies appeared during the development of the models experts discussed the probability values until group consensus was reached.

PT (Probability Tables) experts’ estimates were elicited using standardized probability scales as elicitation format. On these scales, point probabilities were expressed in both numerical values and related verbal expressions (“verbal anchors”), such as “impossible” for 0.0 and “certain” for 1.0.

To calculate the conditional probability tables (CPTs), experts were asked to estimate weights for each parent node in per cent. These weights were used to represent how much influence each parent node exerts on the child node.

### 2.1. Step 1: Identification of Relevant Variables and Causal Relationships

Trajectory prediction accuracy is key to get the best quality of the trajectory information and the traffic demand. However, tools and trajectory prediction models will always present a certain level of uncertainty. When modelling aircraft trajectory, there are several factors introducing uncertainty to the trajectory computation model. Identification and characterization of the main sources of trajectory uncertainty, hereafter referred to as complexity generators (CGs), will allow building an uncertainty model for complexity calculations.

The first step in the Bayesian networks construction is the identification of the relevant variables or “complexity generators” and the assessment of the causal relationships among the variables to be represented in the network. The former to be analysed in the process and the latter with the aims of understanding the interdependences between factors influencing the complexity and the uncertainties associates to those factors. Although in a BN model it is not strictly necessary to include directed links following a causal interpretation, it does make the model much more intuitive, easing the process of getting the dependence and independence relations right, and facilitating significantly the process of eliciting the conditional probabilities of the model. Therefore, proper modelling of causal relationships (i.e., the directed links represent causal relations) will be helpful for the model construction.

**Figure 2:**
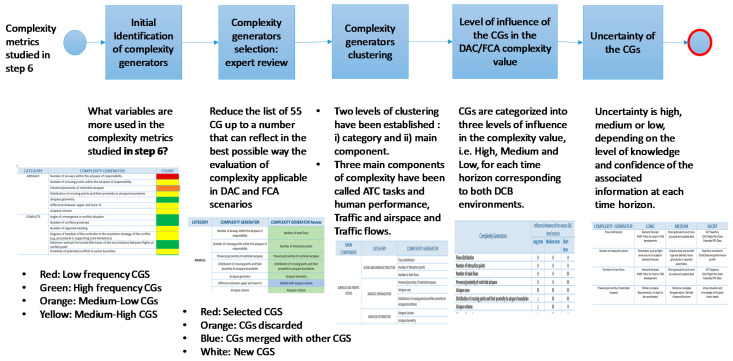
Extracting the Complexity Generators (CGs).

The COTTON (Capacity Optimisation in TrajecTory-based OperatioNs) project approach for the identification of CGs, summarised in Figure 2, is a top-down review of existing complexity metrics combined with a bottom-up analysis of complexity generators in the context of DAC and FC, experts review, clustering and characterization of the level of influence and uncertainty.

Table 2 summarises the list of variables represented in the generic causal model for the complexity uncertainty analysis. Each one of these variables is represented as a node in the network. The list of nodes contains the “complexity generators” (CG), as well as some “intermediate variables” (IV) used to aggregate and integrate the effects of the complexity generators and to show how they contribute to the uncertainty in complexity. It also contains the “outcome” of the network, i.e., the “complexity”.

**Figure 3:**
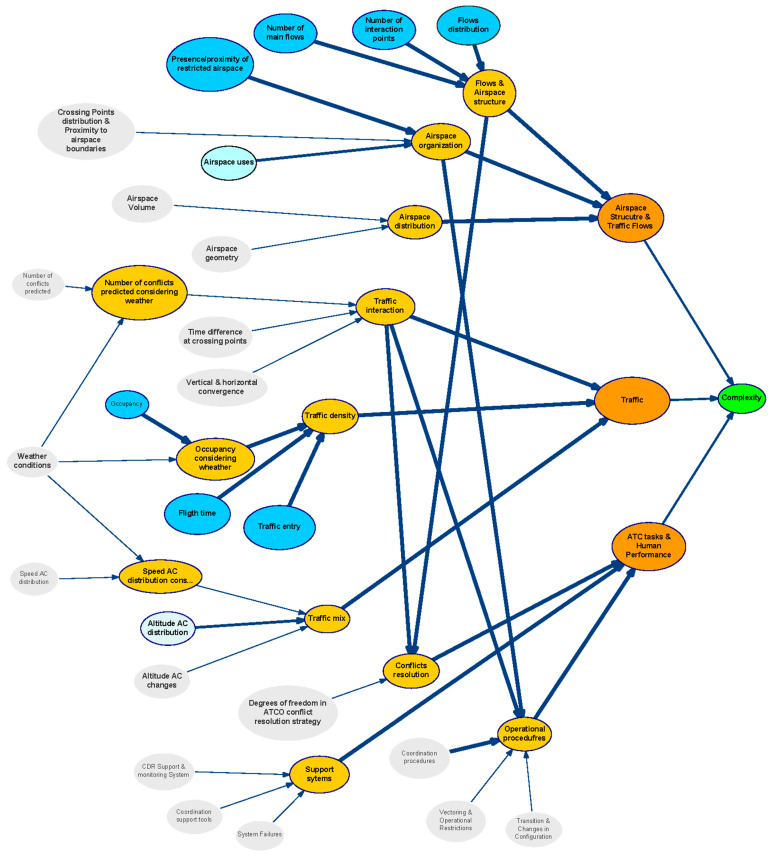
Structure of the generic Bayesian network.

The structure of the generic BN is represented in Figure 3 using the tool GeNIe [50]. In this figure variables are represented as bubbles, and causal relationships are represented as arcs. The thickness of an arc represents the strength of influence between two directly connected nodes. We have used two measures of distance between distributions to validate results: Euclidean and Hellinger [51].

The BN is organised in different layers attending to the nature of the variables (see colours in Figure 3 identifying set of factors). This classification allowed us to understand the causal relationships among influence parameters. The aim is to organise the analysis of the impact for each category when assessing causality and managing uncertainty.

- Blue: Represents the first category of the complexity generators. They are the basic elements that have been considered to contribute to the generation of complexity in a scenario.
- Orange: Indicates the Category of complexity generators; i.e., it groups those complexity generators that may be related to specific elements in an ATM scenario.
- Strong Orange: Identifies the three main components that have been defined (in addition to weather) as final contributors to the complexity of the scenario.

### 2.2. Step 2: State Space of Each Node

The next step in the process is the construction of a state space of the described nodes, i.e., the definition of the variable states and the full joint probability, uniquely defined for all parent nodes in the network. This step implies in our network the discretization of variables, which can be based either on statistical characterization or in expert knowledge. Discretization should provide a balance between losing information and considering an excessive number of states.

**Figure 4:**
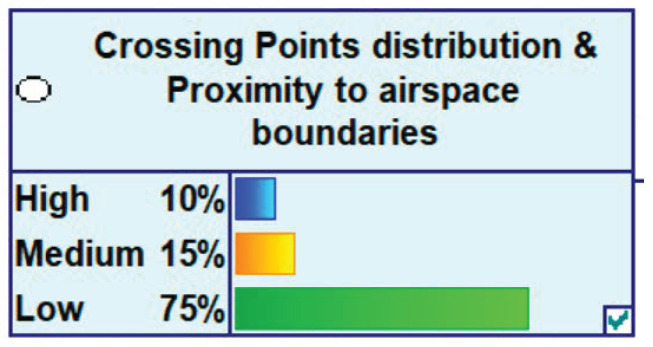
Illustration of the state space of a parent node in the network (probabilities of high, medium and low uncertainty for the variable).

Our model is concerned with the propagation of the uncertainty in the various variables that influence complexity of an ATM scenario. Therefore, the state space of each parent node represents the level of uncertainty that affects a particular variable or complexity generator. The uncertainty affecting each variable has been discretised into three different states: (i) low uncertainty; (ii) medium uncertainty; and (iii) high uncertainty. Therefore, all the nodes have these three states. Then, a probability is allocated, in each node, to each one of these three states, depending on the nature of the variable and the degree of confidence on the information available for each variable at the various time horizons under consideration. The probability distribution at each node represents the probability distribution of the uncertainty of a particular parent node (complexity generator). These three levels are defined by the SESAR Trajectory Based Operations (TBO) environment and by the SESAR Capacity Management processes, Dynamic Airspace Configuration (DAC) and Flight Centric ATC (FCA). Figure 4 illustrates, as an example, how the state space of a parent node is defined in the network.

More precise description of the uncertainty associated to each node could be incorporated in the model, if required, as long as a probability distribution of the uncertainty associated to a particular variable is known. This action will be developed throughout the project, as it progresses in the revision of the corresponding metrics.

The probability, representing the knowledge of the subject before data are gathered, is called “prior” and indicates the likelihood that an input parameter will be in a particular state. When new data or information became available, the prior probability updated and incorporated the evidence into a-posterior probability.

### 2.3. Step 3: Specification of Conditional Probabilities

In the following step, conditional probabilities are specified for non-root nodes for each possible combination of its parent nodes’ values. Conditional probabilities at the child nodes can be obtained by means of statistical learning or from expert knowledge elicitation [52]. In our network, conditional probabilities are derived considering the influence of each variable into its child’s.

Two-different types of nodes are defined in this network: (i) chance binary nodes; and (ii) Noisy-MAX nodes. The output of the network, the node “Complexity” has been specified as a chance binary node with three parent nodes: “Airspace Structure & Traffic Flows”, “Traffic” and “ATC Task & Human Performance”. The rest of the non-root nodes have been defined as noisy-MAX nodes.

### 2.4. Step 3: Reasoning with Bayesian Networks

Due to the conditional dependence relationships of the variables within the network, BNs offer the ability to perform two different type of studies, either predict or diagnose, i.e., they can determine effects and causes. Based on the properties of the Bayesian networks, four different case studies or inferences will be analysed for each of the Bayesian networks.

- **Case study 1:** forward/inter-causal scenario. This model is used to predict the effects; that is, the uncertainty level in the complexity metric (output-child node) by setting the probability of having a certain uncertainties level in the complexity generators, i.e., by setting the probability distribution of the parent-input nodes. This case study is useful to answer the following research question: Given the probability distribution of the uncertainty of the various complexity generators, how do these uncertainties propagate through the network causing a probability distribution for the uncertainty (% of high uncertainty, % of medium uncertainty or % of low uncertainty) in the outcome of the network, “complexity”? This is a typical prediction scenario. Stochastic Sampling is very efficient when there is no evidence in the network. Its complexity is polynomial in the number of nodes.
- **Case study 2:** backward inference. This model is used to deliver a particular configuration in the parent nodes by setting the outcome node (uncertainty level of the complexity metric) to a target value. In this analysis, complexity uncertainty is settled to a high, medium or low value. Then, the network provides understanding about the main contributors to complexity uncertainty, or what configuration of uncertainty might be admitted at the various complexity generators to provide the target outcome uncertainty. This case study is useful to answer the following questions: how much will it be necessary to improve uncertainty in the inputs nodes to achieve a certain uncertainty level in the outcome node? What will be the probability of any fault (uncertainty level of the input nodes) given a set of symptoms or results (uncertainty level of the outcome)? This is a typical fault diagnosis scenario. For backward propagation, we have used Backward Sampling as implemented in GeNie and described in [53].
- **Case study 3:** sensitivity analysis. Sensitivity analysis is used to investigate the effect of small changes in numerical parameters (i.e., probabilities) on the output parameters (e.g., posterior probabilities). Highly sensitive parameters affect the reasoning results more significantly. Identifying them allows for a directed allocation of effort in order to obtain accurate results of a Bayesian Network model. GeNIe implements an algorithm proposed by Kjaerulff and van der Gaag [54], which performs a simple sensitivity analysis in Bayesian networks. Roughly speaking, given a set of target nodes, the algorithm efficiently calculates a complete set of derivatives of the posterior probability distributions over the target nodes over each of the numerical parameters of the Bayesian network. These derivatives give an indication of importance of precision of network numerical parameters for calculating the posterior probabilities of the targets. If the derivative is large for a parameter p, a small deviation in p may lead to a large difference in the posteriors of the targets. If the derivative is small, even large deviations in the parameter make little difference in the posteriors. The results of the sensitivity analysis are presented graphically as indicated in Figure 9. The colouring of the individual elements of the definition shows those individual parameters that are important. Hovering over them shows additional information from which we can read the numerical values of the computed derivatives.
- **Case study 4:** evidence observation. As far as detailed characterization of the uncertainty of a variable is observed (high, medium or low uncertainty), the evidence entered can be visualized as spreading across the network. Then, the network can be updated with such evidence, and the conditional probabilities of the rest of the variables, including the outcome, are recalculated. These new values allow us to evaluate how much improvement can be achieved in reducing the uncertainty of the network outcome by improving the uncertainty related to one or some of the complexity generators. This is also a prediction scenario. This analysis is not included in this report, but it will be performed through the duration of the project as long as the works on complexity algorithms improvement progress. Case study 4 can be considered as an extension of case study 1, where the probability distribution is updated with more precise data.

## 3. DAC Complexity Bayesian Network

In this section, we describe one of the seven models developed as an example of the networks and reasoning performed. The case study used as example corresponds to the Bayesian network for assessing Complexity Uncertainty under the DAC operational framework in the long term horizon.

The model have been constructed from the generic model by discriminating which complexity generators have been considered relevant for each operational concept and time horizon, (long-term, medium term and short term/execution). This information is summarised in Table 3.

As can be observed in columns 2, 3 and 4 of the Table 3, experts have considered that when looking into the DAC complexity at each time horizon, some complexity generators are relevant over complexity results (those marked as H or M), whereas others are not considered relevant (those marked as L).

For example, considering the long term horizon (column 2), only the uncertainties of those factors related with the spatio-temporal interdependencies between the traffics are relevant, whereas the factors related with the operational procedures, organisation of the airspace as well as those related with human factors and performances are not relevant. These factors are considered as having low relevance or influence for assessing complexity uncertainty propagation in this operational concept in the long term, and consequently, are classified as Low (L) in Table 3. Experts have also considered that uncertainty related to a few factors has medium relevance in the long term and uncertainty related to other factors has high relevance. Those factors are indicated as M and H respectively in the second column in Table 3.

The Bayesian network for the DAC concept at each time frame is constructed by filtering all the complexity generators that are not considered relevant or present in that time horizon, (which are basically those indicated as having Low (L) influence or relevance in the corresponding column in Table 3), from those having medium or high relevance. In probabilistic terms the influence of a parent node uncertainty (L, M, H) determines how this parent node affects the probability distribution of its child’s. A parent node having Low influence means that, irrespectively of the level of uncertainty affecting this variable, it has no influences in the child nodes, so such uncertainty will not be propagating to its childs nodes through the network; and therefore the uncertainty of the child does not depend on the uncertainty of the parent node. A parent node having High influence means that the level of uncertainty affecting this variable will propagate to its childs nodes trough the network.

Additionally, Table 4, presents the marginal probability distribution specified from the experts inputs and feedback, for each complexity generator uncertainty at each time horizon of the DAC concept.

It is necessary to emphasize that these values have been taken initially to develop the model and obtain the first results. They try to reflect a qualitative estimate of the level of uncertainty that each of the complexity generators could have, thus, the important thing is not the value, but the assignment of the high/medium/low uncertainty. Throughout the project, this distribution will be reviewed and the estimation improved, depending on the progress of the project.

The combination of Table 3 and Table 4 defines univocally the Bayesian network for complexity analysis at each time horizon.

### 3.1. Description of the Network

**Figure 5:**
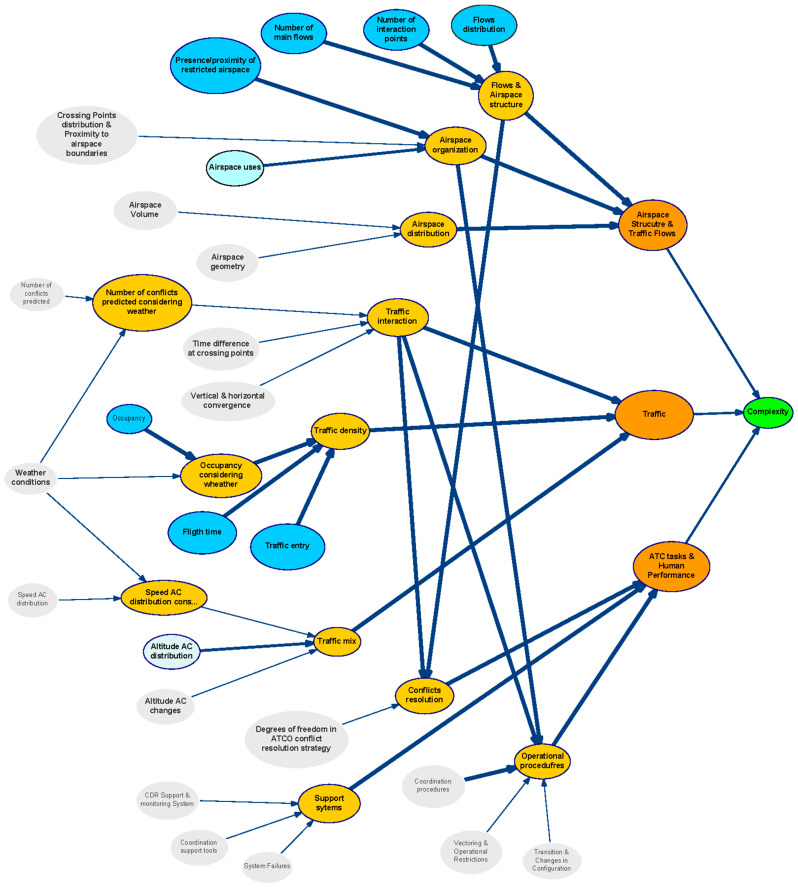
Bayesian network for assessing Complexity Uncertainty under DAC operational framework in the long term horizon (DAC-long model).

Figure 5 presents the resulting network after applying the previous criteria. In this figure, variables are represented as bubbles, and causal relationships are represented as arrows. Bubbles in grey colour indicates that the uncertainty in that node has very Low (L) influence or relevance in the network; bubbles in light blue indicates that uncertainty in that node has Medium (M) influence or relevance in the network, and finally bubbles in dark blue colour indicates that the node has High (H) influence or relevance in the network. Intermediate nodes are indicated in yellow and orange, and the outcome node is indicated in green. In this figure, the thickness of an arrow represents the strength of influence between two directly connected nodes. According to this the complexity generators with higher influence are:

- Presence/proximity of restricted airspace;
- Occupancy;
- Flows distribution;
- Number of interaction points;
- Number of main flows;
- Flight times;
- Traffic entries

As can be observed, certain variables have been considered to have low impact in the DAC complexity uncertainty for the long timeframe:

- Distribution of crossing points and their proximity to airspace boundaries;
- Airspace volume;
- Airspace Geometry;
- Speed AC distribution;
- Altitude AC changes;
- Number of conflicts predicted;
- Time difference at crossing points;
- Vertical and horizontal convergence (diverging, constant or converging);
- Coordination procedures;
- Vectoring and operational restrictions;
- Transition and changes in configuration;
- Degrees of freedom of the controller in the resolution strategy of the conflict (e.g., procedural or supporting tools limitations);
- CDR Support and monitoring System;
- Coordination support tools;
- System failure;
- Weather conditions

That means that whatever the state of the uncertainty would be at these variables, it will have very little impact on the complexity uncertainty and it will barely propagate trough the network.

### 3.2. Analysis of the DAC Long Term BN Scenarios

#### 3.2.1. Forward/Inter-Causal Scenario

**Figure 6:**
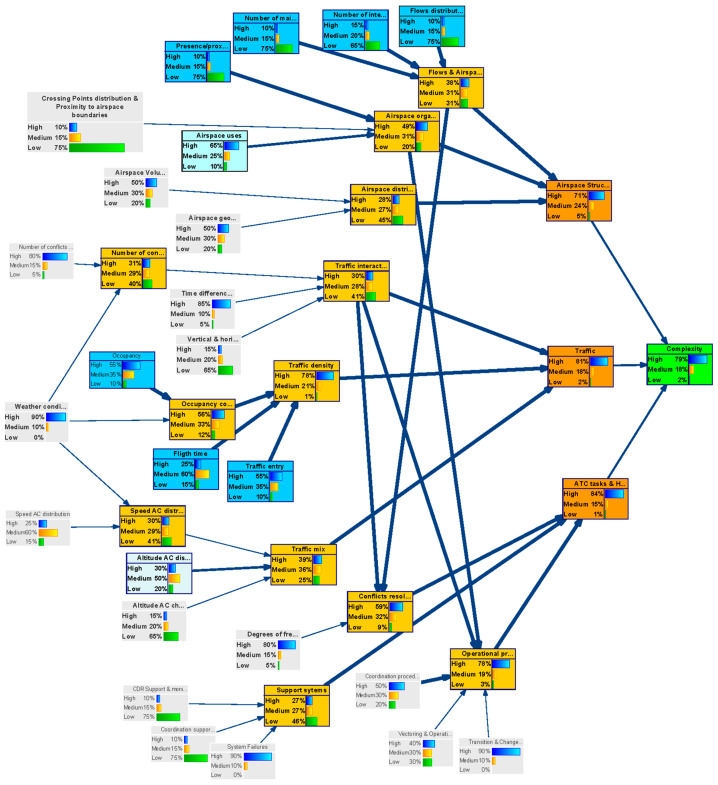
Forward inter-causal analysis (DAC long model).

In this case study, the network is used to predict the effects, given the inputs. Figure 6 shows the uncertainty level in the complexity metric (output-child node) by setting the probability of having a certain uncertainties level in the complexity generators (as defined by the experts), i.e., by setting the probability distribution of the parent-input nodes.

Given the probability distribution of the uncertainty of the various complexity generators, the figure illustrates how these uncertainties propagate through the network causing a probability distribution for the uncertainty (% of high uncertainty, % of medium uncertainty or % of low uncertainty) in the outcome of the network, “complexity”. It can be shown that in the long term timeframe in the DAC concept, considering uncertainty in the inputs estimated by the experts, the probability of the indicator “complexity” having:

- High uncertainty will be 79%.
- Medium uncertainty will be 18%.
- Low uncertainty will be 3%.

For this reason, it is necessary to identify the complexity generators that should improve their level of uncertainty in order to achieve more reliable metrics. This is the analysis performed in the following case.

#### 3.2.2. Backward Inference

In the backward analysis, the model is used to obtain a particular configuration of the parent nodes states by setting the outcome node (uncertainty level of the complexity metric) to a target value.

This analysis helped us to identify by how much it will be necessary to improve uncertainty in the input nodes to achieve a particular level of uncertainty in the outcome node–“complexity”.

**Figure 7:**
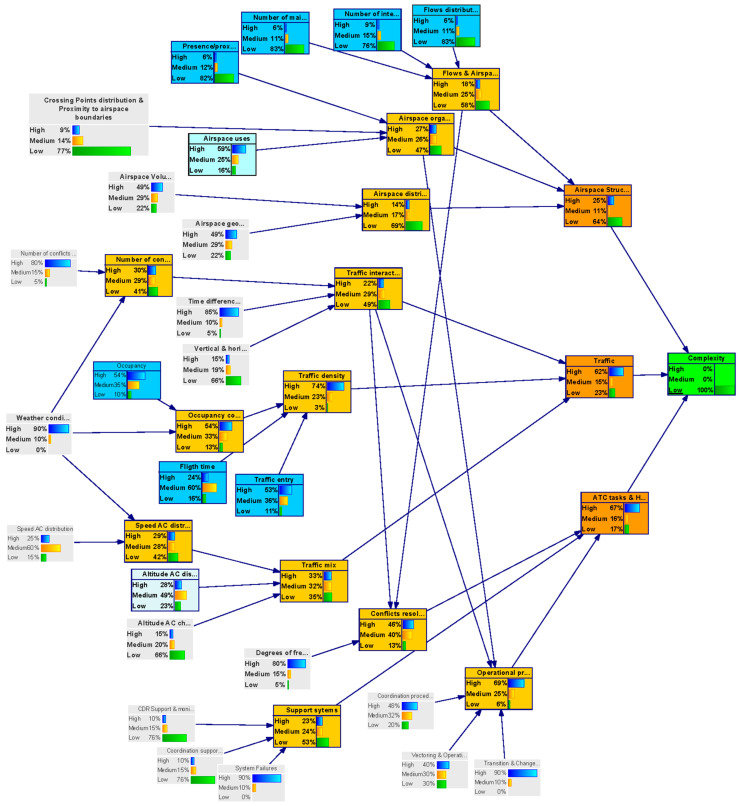
Backward inference for low complexity (DAC-long model).

**Figure 8:**
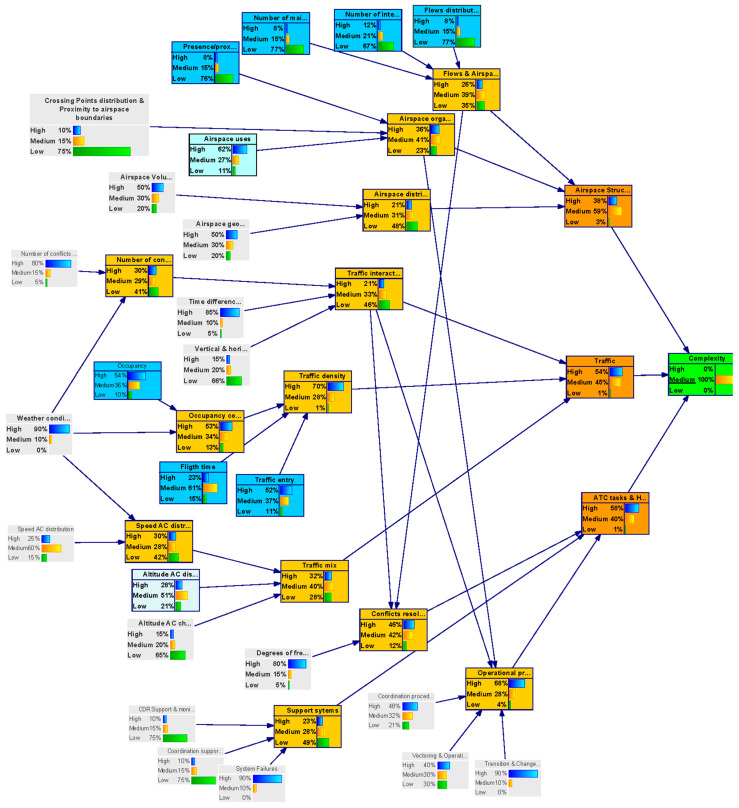
Backward inference for medium complexity (DAC-long model).

In total, three backward inference analysis were performed:

(i) Complexity uncertainty is settled to a Low value with 100% probability (see Figure 7);
(ii) Complexity uncertainty is settled to a Medium value with 100% probability (see Figure 8);
(iii) Complexity uncertainty is settled to a High value with 100% probability.

The results of the three analyses were combined and integrated in Table 5. The rows indicate the target value set for the complexity node in the backward analysis (Low uncertainty, Medium, uncertainty and High uncertainty, respectively). The columns indicate the state the parent variables take in the backward analysis. The colour indicates the influence of a node in the network output— “complexity”. Nodes with low influence are shadowed in green, nodes with medium influence are shadowed in blue and nodes with high influence are shadowed in red.

#### 3.2.3. Sensitivity Analysis

**Figure 9:**
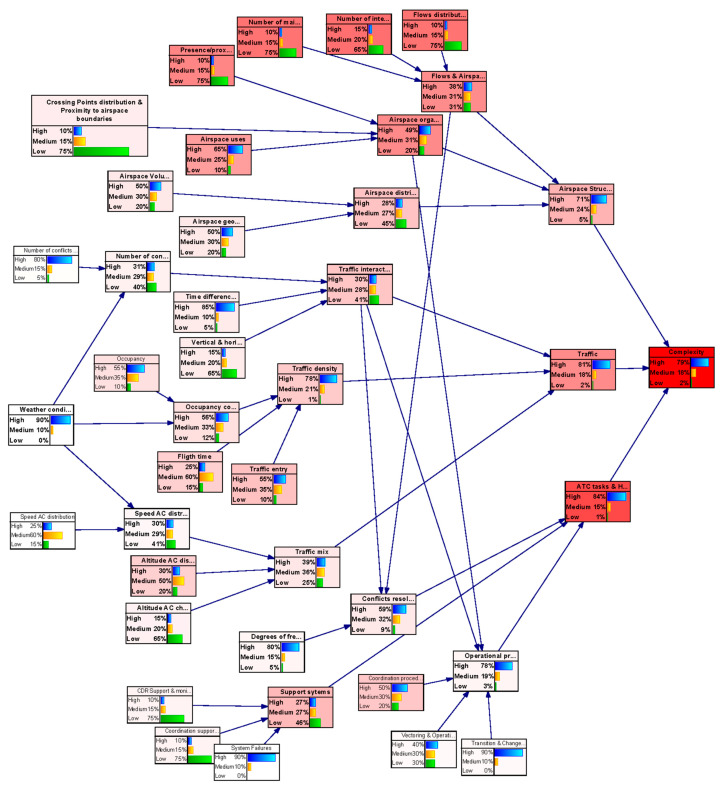
Sensitivity analysis (DAC-long model).

Figure 9 presents the result of the sensitivity analysis. It highlights, in different shade of red, the nodes in the network which are the parameters that affect the reasoning results more significantly. The results bear out the outcomes from the previous analysis, highlighting the complexity generators that contribute to a greater extent to maintain a low uncertainty in the complexity result, and those whose variation does not affect the uncertainty in the final result.

## 4. Discussion of Results

**Figure 10:**
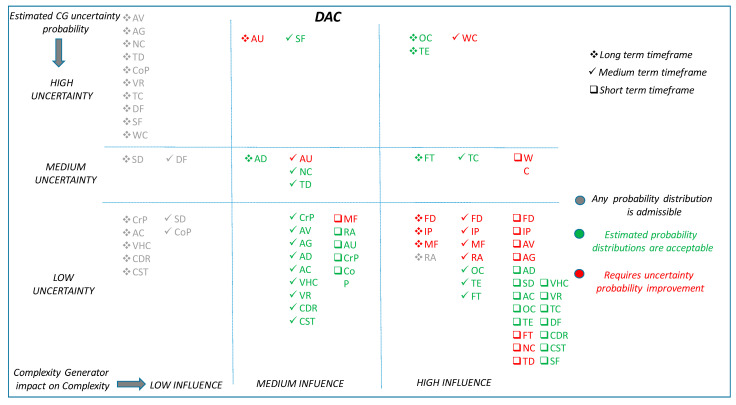
Overall situation for Complexity Generators. DAC application.

**Figure 11:**
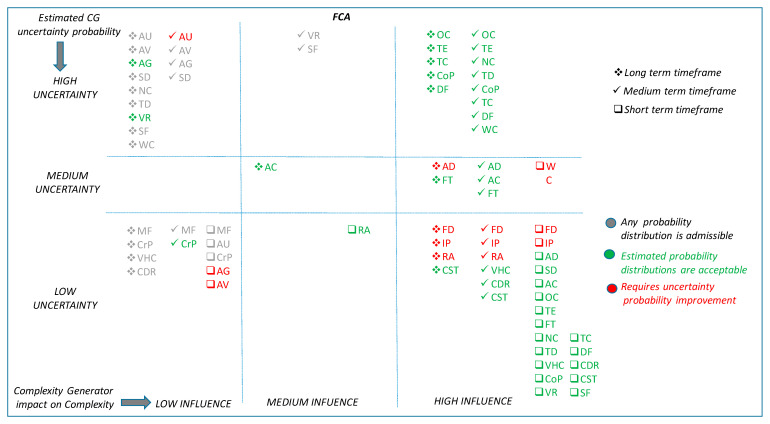
Overall situation for Complexity Generators. FCA application.

Figure 10 and Figure 11 summarise, in a multidimensional diagram, the conclusions derived from the BN case studies. In order to facilitate the understanding of these figures, the rationality behind both of them is explained hereafter. The diagram is divided into nine regions, where complexity generators are classified according to its influence on complexity and its degree of uncertainty:

Level of influence on complexity. This is shown in the horizontal dimension (rows) of the diagram. Complexity generators in the first column have high influence in the complexity, those in the second column have medium influence and the ones in the third column have low influence on complexity.

Degree of uncertainty of the information. This is shown in the vertical dimension of the diagram. Complexity generators in the first row suffer from high uncertainty, complexity generators in the second row have medium uncertainty and the ones in the third row are known to have low uncertainty.

For example, complexity generators placed on the bottom right corner of the diagram have high influence on complexity and a low degree of uncertainty associated to its information.

Each one of the time horizons considered is reflected with a different symbol: □ Short Term; √ Medium Term; * Long Term.

Additionally, the colour code reflects the results of the analysis:

- Red: indicates that the uncertainty of a complexity generator, for the considered timeframe, needs to be improved (i.e., reduced).
- Green: indicates that the uncertainty of a complexity generator, for the considered timeframe, may be acceptable.
- Grey: indicates that the uncertainty of a complexity generator, for the considered timeframe, does not affect the final result due to the low influence of the variable.

Complexity generators that have a high influence on “complexity” should conform the pool of preferred candidates to be measured, quantified and evaluated by the complexity metrics and algorithms. These are placed in the right hand side column of the multidimensional diagram. Complexity generators with low influence were considered a-priori less suitable parameters for the complexity metrics and algorithms. They were placed in the left hand side column of the multidimensional diagram. Finally, those with medium influence might deserve detailed and individual consideration to evaluate up to what extent they contribute to the complexity algorithms and metrics, depending upon the demand and capacity balance application and timeframe, and the uncertainty with which each variable is known. These are located in the central column of the multidimensional diagram.

Table 6 shows the acronyms code used in the figure to represent each complexity generator.

In relation to the results established in Figure 10, the following conclusions are drawn for the DAC application.

As a final summary, Table 7 presents the set of complexity generators recommended as inputs in the complexity metrics and algorithms for each application and time horizon, highlighting in red colour those that would require further reduction in uncertainty.

## 5. Conclusions

The main objective of the COTTON project was to deliver innovative solutions to maximize the effectiveness of the Capacity Management processes taking full advantage of the trajectory information available in a TBO environment. In this context, the main objective of this paper was to address the challenge of exploring how the uncertainties associated with the agreed trajectory will influence the quality of the predictions of complexity of traffic demand and the effectiveness of DCB processes regarding airspace management.

To achieve this aim, we have developed and implemented an ad-hoc methodology combining qualitative and quantitative approaches that integrates state of the art of SESAR works on complexity, experts knowledge and advanced causal and predictive BN models.

Causal predictive models have been developed, using Bayesian Networks, to evaluate the effect of trajectory uncertainty on complexity assessment. The aim of the networks is to help to identify the relevant variables in the process, and to understand the causal relationships and interdependences between factors influencing the complexity and the uncertainties associated to those factors. In total, seven BN models have been elicited covering each concept and time horizon.

The outcome of these BN models was double. On the one hand, they will help to identify what complexity generators need to be properly taken into account and analysed by the complexity algorithms and metrics adapted to the DAC and FCA concepts. Among the set of current variables considered complexity generators, the BN will help to highlight which ones are expected to have a High (H), Medium (M) or Low (L) influence in the complexity outcome itself, depending on the concept and timeframe considered. The future complexity algorithms and metrics might take this outcome as an indication of which variables, because of their influence on complexity, need to be carefully addressed by the metrics and algorithms. On the other hand, the networks will help to identify whether the a-priori uncertainty probability distribution of each of those variables (considering the various timeframe horizons) will be compatible with maintaining the uncertainty of complexity outcome at a LOW or MEDIUM state, or which ones will need further improvements reducing its level of uncertainty. The future complexity algorithms and metrics might take this outcome as an indication of the uncertainty they might expect for each complexity generator (High, Medium or Low), and according to that decide the best approach to characterise and measure this variable in each scenario.

As a result of all this work, the main achievements of the research can be summarised as follows:

- Assessment of how trajectory uncertainty is translated into complexity uncertainty.
- Limitations of existing complexity assessment methods to address the challenges of SESAR 2020 Capacity Management processes (DAC and FCA).
- For each application (DAC or FCA) and different time horizons, identification of the Complexity Generators that generate greater uncertainty in the complexity results. Similarly, if a certain level of uncertainty is required for the complexity methods, taking into account the conditions of application of the TBO concept, identification of the set of complexity generators recommended for a given scenario.
- A pool of preferred candidates to be used as complexity generators; that is, the set of complexity generators recommended as inputs in the complexity metrics and algorithms for each application and time horizon.
- The list complexity generator that would require further reduction in uncertainty.
- An evaluation of the adaptability of current complexity metrics for DAC and FCA environment.

Future work will consist of improving the models with real data. The models will be used through the different phases of the COTTON project according to the precision and accuracy of the information available to characterise uncertainty associated to the complexity generators and to the complexity metric.

In an early phase of the project, there were no data available for the characterisation of the uncertainty of the variables, and the information to characterise variables uncertainty and their impact on complexity was either qualitative or elicited from expert knowledge. Therefore, the network, although it is able to provide numerical figures, has to be used as a qualitative cause and effect indication, considering the trends instead of the numerical values. During the project evolution, as metrics will be improved to deal with uncertainty in DAC and FCA environments, the data obtained about its performance will be fed into the Bayesians models, updating the probability distributions and making them more accurate. Given a situation and a prior probability distribution over a hypothesis variable that represents possible courses of action, Bayesian network provides the capability to update this probability distribution when fresh data and information are obtained. That way, depending upon the quality and availability of the data, the revised probability distribution will allow to perform more quantitative analyses.

## Figures and Tables

**Table 1:** Bayesian Network models developed.

Bayesian Models Developed		
**A generic Bayesian network for complexity**	Three Bayesian networks for DAC complexity, representing: Complexity for DAC in the long term horizon. Complexity for DAC in the medium term horizon. Complexity for DAC in the short term/execution horizon	Three Bayesian networks for FCA complexity, representing: Complexity for FCA in the long term horizon. Complexity for FCA in the medium term horizon. Complexity for FCA in the short term/execution horizon.

Notes: DAC: Dynamic Airspace Configuration; FCA: Flight Centric Air Traffic Control.

**Table 2:** List of variables represented in the causal model (nodes at the Bayesian network).

Node Type	Node: Uncertainty Level in the Variable	Node Type	Node: Uncertainty Level in the Variable
CG 1	Flows distribution	CG 2	Number of interactions points
CG 3	Number of main flows	CG 4	Presence/proximity of restricted airspace
CG 5	Crossing points distribution & proximity to airspace distribution	CG 6	Airspace uses
CG 7	Airspace volume	CG 8	Airspace geometry
CG 9	Number of conflicts predicted	IV 1	Number of conflicts predicted considering weather
CG 10	Time difference at crossing points	CG 11	Vertical & horizontal convergence
CG 12	Occupancy	IV 2	Occupancy considering weather
CG 13	Distribution of flight time per aircraft	CG 14	Traffic entry
CG 15	Speed AC distribution	IV 3	Speed AC distribution considering weather
CG 16	Weather	CG 17	Altitude AC distribution
CG 18	Altitude AC changes	CG 19	Degrees of freedom in ATCO (Air Traffic Controller) conflict resolution strategy
CG 20	CDR Support & monitoring System	CG 21	Coordination Support Tools
CG 22	System failures	CG 23	Coordination procedures
CG 24	Vectoring & Operational Restrictions	CG 25	Transition and changes in configuration
IV 4	Flows & Airspace structure	IV 5	Airspace Organisation
IV 6	Airspace Distribution	IV 7	Traffic Interaction
IV 8	Traffic Density	IV 9	Traffic mix
IV 10	Conflicts resolution	IV 11	Support systems
IV 12	Operational procedures	IV 13	Airspace Structure & Traffic Flows
IV 14	Traffic	IV 15	ATC tasks six human Performance
Outcome	Complexity		

**Table 3:** Relevance of each complexity generator at the various DAC time horizons.

Complexity Generators	Influence/Relevance at the Various DAC Time Horizons		
	Long Term	Medium Term	Short Term
Flows distribution	H	H	H
Number of interaction points	H	H	H
Number of main flows	H	H	M
Presence/proximity of restricted airspace	H	H	M
Airspace uses	M	M	M
Distribution of crossing points and their proximity to airspace boundaries	L	M	M
Airspace volume	L	M	H
Airspace Geometry	L	M	H
Altitude AC distribution	M	M	H
Speed AC distribution	L	L	H
Altitude AC changes	L	M	H
Occupancy	H	H	H
Traffic entry	H	H	H
Flight time	H	H	H
Number of conflicts predicted	L	M	H
Time difference at crossing points	L	M	H
Vertical and horizontal convergence (diverging, constant or converging)	L	M	H
Coordination procedures	L	L	M
Vectoring and operational restrictions	L	M	H
Transition and changes in configuration	L	H	H
Degrees of freedom of the controller in the resolution strategy of the conflict (e.g., procedural or supporting tools limitations)	L	L	H
CDR Support and monitoring System	L	M	H
Coordination’s support tools	L	M	H
System failure	L	M	H
Weather conditions	L	H	H

**Table 4:** Specification of Marginal Probability distribution for the parent nodes.

Complexity Generators	States	Distribution of Probability for Each Uncertainty State of the Parent Nodes or Complexity Generators (%)		
		Long Term Horizon	Medium Term Horizon	Short Term Horizon
**Flows distribution**	H	0.1	0.05	0
	M	0.15	0.1	0.05
	L	0.75	0.85	0.95
**Number of interaction points**	H	0.15	0.1	0
	M	0.2	0.15	0.05
	L	0.65	0.75	0.95
**Number of main flows**	H	0.1	0.05	0
	M	0.15	0.1	0.05
	L	0.75	0.85	0.95
**Presence/proximity of restricted airspace**	H	0.1	0.05	0
	M	0.15	0.1	0.01
	L	0.75	0.85	0.99
**Airspace uses**	H	0.65	0.2	0
	M	0.25	0.55	0.01
	L	0.1	0.25	0.99
**Distribution of crossing points and their proximity to airspace boundaries**	H	0.1	0.05	0
	M	0.15	0.1	0.05
	L	0.75	0.85	0.95
**Airspace volume**	H	0.5	0.1	0
	M	0.3	0.25	0.05
	L	0.2	0.65	0.95
**Airspace Geometry**	H	0.5	0.1	0
	M	0.3	0.25	0.05
	L	0.2	0.65	0.95
**Altitude AC distribution**	H	0.3	0.1	0.05
	M	0.5	0.25	0.05
	L	0.2	0.65	0.9
**Speed AC distribution**	H	0.25	0.1	0.05
	M	0.6	0.35	0.1
	L	0.15	0.55	0.85
**Altitude AC changes**	H	0.15	0.05	0
	M	0.2	0.15	0.1
	L	0.65	0.8	0.9
**Occupancy**	H	0.55	0.1	0.05
	M	0.35	0.3	0.05
	L	0.1	0.6	0.9
**Traffic Entry**	H	0.55	0.1	0.05
	M	0.35	0.3	0.05
	L	0.1	0.6	0.9
**Distribution of flight time per aircraft under ATCO responsibility in the given timeframe**	H	0.25	0.1	0.05
	M	0.6	0.25	0.05
	L	0.15	0.65	0.9
**Number of conflicts predicted**	H	0.8	0.35	0.05
	M	0.15	0.5	0.15
	L	0.05	0.15	0.8
**Time difference at crossing points**	H	0.85	0.4	0.05
	M	0.1	0.5	0.15
	L	0.05	0.1	0.8
**Vertical and horizontal convergence (diverging. constant or converging)**	H	0.15	0.1	0
	M	0.2	0.15	0.05
	L	0.65	0.8	0.95
**Coordination procedures**	H	0.5	0.1	0
	M	0.3	0.25	0.05
	L	0.2	0.65	0.95
**Vectoring and operational restrictions**	H	0.4	0.1	0
	M	0.3	0.15	0.05
	L	0.3	0.75	0.95
**Transition and changes in configuration**	H	0.9	0.2	0.1
	M	0.1	0.5	0
	L	0	0.3	0.9
**Degrees of freedom of the controller in the resolution strategy of the conflict (e.g., procedural or supporting tools limitations)**	H	0.8	0.2	0.1
	M	0.15	0.5	0
	L	0.05	0.3	0.9
**CDR Support and monitoring System**	H	0.1	0.05	0
	M	0.15	0.1	0.05
	L	0.75	0.85	0.95
**Coordination support tools**	H	0.1	0.05	0
	M	0.15	0.1	0.05
	L	0.75	0.85	0.95
**System failure**	H	0.9	0.8	0
	M	0.1	0.15	0.15
	L	0	0.05	0.85
**Weather conditions**	H	0.9	0.8	0.35
	M	0.1	0.2	0.45
	L	0	0	0.2

**Table 5:** Summary of the backward inference (DAC-long model).

Target Value Set for Complexity at Each Backward Analysis	States Taken by the Parent Nodes in the Backward Analysis		
	Low Uncertainty State	Medium Uncertainty State	High Uncertainty State
**Low**	Flows distribution Number of interaction points Number of main flows Presence/proximity of restricted airspace Airspace uses Distribution of crossing points and their proximity to airspace boundaries. Vertical and horizontal convergence (diverging, constant or converging) Altitude AC changes CDR Support and monitoring System. Coordination support tools.	Distribution of flight time per aircraft under ATCO responsibility in the given timeframe Speed AC distribution Altitude AC distribution	Airspace volume Airspace Geometry Number of conflicts predicted Weather Occupancy Time difference at crossing points Time difference at crossing points Traffic entry Weather Degrees of freedom of the controller in the resolution strategy of the conflict (e.g., procedural or supporting tools limitations) System failure Coordination procedures Vectoring and operational restrictions Transition and changes in configuration
**Medium**	Flows distribution Number of interaction points Number of main flows Presence/proximity of restricted airspace Airspace uses Distribution of crossing points and their proximity to airspace boundaries. Vertical and horizontal convergence (diverging, constant or converging) Altitude AC changes CDR Support and monitoring System Coordination support tools	Distribution of flight time per aircraft under ATCO responsibility in the given timeframe Speed AC distribution Altitude AC distribution	Airspace volume Airspace Geometry Number of conflicts predicted, weather, occupancy, time difference at crossing points Time difference at crossing points Occupancy Traffic entry Weather Degrees of freedom of the controller in the resolution strategy of the conflict (e.g., procedural or supporting tools limitations) System failure Coordination procedures Vectoring and operational restrictions Transition and changes in configuration

**Table 6:** Codes for Complexity Generators.

Complexity Generator	Code	Complexity Generator	Code
Flows distribution	FD	Distribution of flight time per aircraft under ATCO responsibility in the given timeframe	FT
Number of interaction points	IP	Number of conflicts predicted	NC
Number of main flows	MF	Time difference at crossing points	TD
Presence/proximity of restricted airspace	RA	Vertical and horizontal convergence	VHC
Airspace uses	AU	Coordination procedures	CoP
Distribution of crossing points and their proximity to airspace boundaries	CrP	Vectoring and operational restrictions	VR
Airspace volume	AV	Transition and changes in configuration	TC
Airspace Geometry	AG	Degrees of freedom of the controller in the resolution strategy of the conflict	DF
Altitude AC distribution	AD	CDR Support and monitoring System	CDR
Speed AC distribution	SD	Coordination support tools	CST
Altitude AC changes	AC	System failure	SF
Occupancy (per ATCO position)	OC	Weather conditions	WC
Traffic Entry (per ATCO position)	TE		

**Table 7:** Complexity generators recommended as inputs in the complexity metrics and algorithms for each application and time horizon.

Time Horizon	DAC Recommendation	FCA Recommendation
**Long Term**	Flows distribution Number of interaction points Number of main flows Presence/proximity of restricted airspace Distribution of flight time per aircraft under ATCO responsibility in the given timeframe Airspace uses	Flows distribution Number of interaction points Presence/proximity of restricted airspace Altitude AC distribution Distribution of flight time per aircraft under ATCO responsibility in the given timeframe Airspace uses Coordination support tools.
**Medium Term**	Flows distribution Number of interaction points Number of main flows Presence/proximity of restricted airspace Occupancy (per ATCO position) Traffic Entry (per ATCO position) Transition and changes in configuration	Flows distribution Number of interaction points Presence/proximity of restricted airspace Distribution of flight time per aircraft under ATCO responsibility in the given timeframe Airspace uses Altitude AC distribution Altitude AC changes Vertical and horizontal convergence Coordination support tools. CDR Support and monitoring System
**Short Term**	Flows distribution Number of interaction points Airspace volume Airspace Geometry Distribution of flight time per aircraft under ATCO responsibility in the given timeframe Number of conflicts predicted Time difference at crossing points Weather conditions Altitude AC distribution Speed AC distribution Altitude AC changes Occupancy (per ATCO position) Traffic Entry (per ATCO position) Vertical and horizontal convergence Vectoring and operational restrictions Transition and changes in configuration Degrees of freedom of the controller in the resolution strategy of the conflict CDR Support and monitoring System Coordination support tools System failure	Flows distribution Number of interaction points Weather conditions Altitude AC distribution Speed AC distribution Altitude AC changes Occupancy (per ATCO position) Traffic Entry (per ATCO position) Distribution of flight time per aircraft under ATCO responsibility in the given timeframe Number of conflicts predicted Time difference at crossing points Vertical and horizontal convergence Coordination procedures Vectoring and operational restrictions Transition and changes in configuration Degrees of freedom of the controller in the resolution strategy of the conflict CDR Support and monitoring System Coordination support tools System failure
